# Development and Characterization of New Monoclonal Antibodies against Human Recombinant CA XII

**DOI:** 10.1155/2014/309307

**Published:** 2014-05-20

**Authors:** Dovile Dekaminaviciute, Rita Lasickiene, Seppo Parkkila, Vaida Jogaite, Jurgita Matuliene, Daumantas Matulis, Aurelija Zvirbliene

**Affiliations:** ^1^Institute of Biotechnology, Vilnius University, V. A. Graiciuno 8, 20241 Vilnius, Lithuania; ^2^School of Medicine and Institute of Biomedical Technology, University of Tampere and Fimlab Ltd., Medisiinarinkatu 3, 33520 Tampere, Finland

## Abstract

Carbonic anhydrases (CAs) are enzymes that catalyse the reversible hydration of CO_2_ to bicarbonate. CA XII is considered a potential biomarker of tumor cells and a promising target for specific therapies. The aim of the current study was to develop new monoclonal antibodies (MAbs) against human recombinant CA XII and evaluate their diagnostic potential. An extracellular catalytic domain of human CA XII was expressed in *E. coli* and used as an immunogen. Seven stable hybridoma cell lines producing high-affinity IgG antibodies against human CA XII were generated. The majority of MAbs were highly specific to CA XII and did not cross-react with human recombinant CA I, CA II, CA VII, and CA XIII. In order to demonstrate the diagnostic value of the MAbs, they were employed for the immunohistochemistry analysis of CA XII expression in tissues. Two MAbs (15A4 and 4A6) demonstrated a strong and specific immunostaining of CA XII in human tissue specimens. Flow cytometry analysis of 5 human tumor cell lines with the MAb 15A4 revealed its immunoreactivity with cellular CA XII. In conclusion, the MAbs raised against recombinant catalytic domain of CA XII recognize cellular CA XII and represent a promising diagnostic tool for the immunodetection of CA XII-expressing cells.

## 1. Introduction

The *α*-carbonic anhydrases (*α*-CAs, EC 4.2.1.1) belong to metalloenzymes that catalyse the reversible hydration of carbon dioxide to bicarbonate (H_2_O + CO_2_ *↔* H^+^ + HCO_3_^−^). So far, 15 different human CAs are identified that differ in their enzymatic properties, subcellular localization, and tissue distribution. Enzymatically active human CAs are either cytosolic (CA I, CA II, CA III, CA VII, and CA XIII), membrane bound (CA IV, CA IX, CA XII, and CA XIV), mitochondrial (CA VA, and CA VB), or secretory (CA VI). CAs are expressed in various cells and tissues such as erythrocytes, gastrointestinal tract, reproductive tract, the nervous system, kidney, lung, skin, eye, and muscle [1–6]. The most important function of CAs is the transport of CO_2_ in various metabolizing tissues. CAs are also involved in other physiological functions: pH regulation, ion transport, bone resorption and secretion of gastric juice, cerebrospinal fluid, and pancreatic juice [4].

Recent studies indicate that at least two of CA transmembrane isozymes—CA IX and XII—are associated with human cancers. CA IX is a well-recognized tumor marker as it is overexpressed in many types of tumors and is found in only a few normal tissues [2]. There are increasing evidences on the potential of CA XII as a new tumor marker. CA XII is a transmembrane protein with an extracellular catalytic domain. CA XII overexpression has been demonstrated in human renal cell carcinoma [7] as well as in brain, colorectal, breast, gastrointestinal, ovarian, and pancreatic cancers [6]. CA XII is also expressed in the normal human kidney, colon, lung, brain, prostate, ovary, and testis [1, 8].

The expression of CA IX and CA XII is induced under hypoxic conditions through hypoxia inducible factor-1. Hypoxia and consequent acidosis of tumor microenvironment are principal features of many types of solid cancers. Both CA IX and CA XII promote tumor growth and survival through pH maintenance [6, 8–10]. Recent studies suggest that the functions of CA IX and CA XII related to tumor growth and metastasis, as well as their membrane-associated localization, make these enzymes promising targets for specific therapies. Sulfonamides represent the main group of the specific CA chemical inhibitors [11, 12]. Two monoclonal antibodies (MAbs) against CA IX were generated as specific immunological tools for clinical detection and therapy. Their diagnostic value has been confirmed by immunohistochemistry and radiolabeled monoclonal antibody imaging [9]. Recently, the importance of CA XII as a serodiagnostic marker for lung cancer has been demonstrated [13]. The MAb 6A10 raised against CA XII expressed in lung cancer cells has been shown to inhibit CA XII enzymatic activity and tumor growth* in vitro* [14].

The aim of the current study was to develop new monoclonal antibodies (MAbs) against human recombinant CA XII and evaluate their diagnostic potential. We have demonstrated that the MAbs raised against recombinant catalytic domain of CA XII recognize cellular CA XII and are valuable reagents for its immunodetection in human tumor tissue specimens.

## 2. Materials and Methods

### 2.1. Production of Recombinant Carbonic Anhydrases

Recombinant extracellular domain of human CA XII spanning amino acid (aa) residues from 30 to 291 was expressed in* E. coli* cells and purified as described previously [15]. In brief,* E. coli* Rosetta (DE3) strain cells (Novagen, Germany) were transformed with recombinant expression vector pET21a-CA XII. Transformants were cultured in Luria-Bertani (LB) medium, containing 100 *μ*g/mL ampicillin and 34 *μ*g/mL chloramphenicol and grown at 37°C and 220 rpm for 16 h. The expression of CA XII was induced with 1 mM isopropyl b-D-thiogalactoside (IPTG) and in the presence of 0.5 mM ZnSO_4_. The culture was grown for 4 h at 30°C and 220 rpm. The cells were harvested, mixed with lysis buffer (20 mM Hepes, 0.1% Triton X-100, 0.15 M NaCl, and 1 mM PMSF; pH 8.5), and disrupted by sonication. The soluble protein fraction was purified using a CA-affinity column containing p-(aminomethyl)benzenesulfonamide agarose (Sigma-Aldrich, St. Louis, USA). Eluted CA XII protein was dialyzed against a storage buffer containing 10 mM Hepes (pH 7.5) and 50 mM NaCl. To test the specificity of the MAbs to glycosylated form of CA XII, the recombinant extracellular domain of human CA XII was expressed in human cell line HEK293. For this purpose, expression plasmid based on pCEP4dS vector designed for the secretion of recombinant mammalian proteins was constructed. The pCEP4dS vector for insertion of CA XII gene was constructed from the pCEP4 vector (Invitrogen, Life Technologies) by making two modifications. First, the vector size was reduced by the deletion of 1990 base pair fragment between the restriction sites of SalI (9953) and NruI (7963). Second, the secretion signal was introduced into a multiple cloning sites. Two complementary single stranded oligonucleotides, containing the secretion signal from the V-J2-C region of murine Ig kappa chain were chemically synthesized and annealed. The resultant double stranded oligonucleotide was digested at the 5′ and 3′ ends with KpnI and HindIII, respectively, and ligated into the vector cut with the same enzymes. For the expression of mammalian CA XII, the pCEP4dS-CAXII plasmid was constructed. The DNA fragment, corresponding to the catalytic domain of CA XII (amino acids 30 to 291), was cut out from the pET21a-CAXII plasmid [15] with NdeI and BamHI restriction endonucleases and ligated into the pCEP4dS vector, digested with HindIII and BamHI. Recessed 3′-termini resulting from the digestion with NdeI and HindIII were filled in by Klenow fragment before the ligation. Because of the linker located between the secretion signal and the coding sequence of CA XII, the expressed CA XII protein has additional 8 amino acids (DAAHMKLM) at the N terminus.

Expression of CA XII was carried out using the FreeStyle Max 293 expression system (Invitrogen, Life Technologies). FreeStyle 293-F suspension cell culture was maintained in 125–500 mL Erlenmeyer flasks containing 30–120 mL of FreeStyle medium in a 37°C incubator with a humidified atmosphere of 8% CO_2_, on an orbital shaker platform rotating at 135 rpm. FreeStyle cells were transiently transfected with the purified pCEP4dS-CAXII plasmid according to manufacturer's recommendations. Four days later, the cell culture was centrifuged at 6000 g for 20 min and the secreted CA XII protein was purified from the supernatant using a CA-affinity column containing p-(aminomethyl)benzenesulfonamide agarose (Sigma-Life Science Aldrich). The eluted CA XII protein was dialyzed into a storage buffer containing 10 mM Hepes (pH 7.5) and 50 mM NaCl and stored at −80°C.

Recombinants CA I, CA II, CA VII, and CA XIII were expressed in* E. coli* and purified as described previously [16].

### 2.2. Production of GST-Fused CA XII Segments for Epitope Mapping

DNA fragments encoding three overlapping segments of CA XII, number 1 (aa 27–130), number 2 (aa 111–210), and number 3 (184–290), were amplified from full length CA XII DNA by polymerase chain reaction (PCR) and cloned into bacterial expression vector pGex4T2. Three pairs of primers with restriction endonuclease recognition sites, start and stop codons, were used in PCR (Table 1).

CA XII segments fused to glutathione-S-transferase (GST) were expressed in* E. coli* BL21 (DE3) strain (Novagen). Transformed cells were grown in LB medium, containing 100 *μ*g/mL ampicillin at 37°C and 220 rpm for 16 h. The saturated culture was diluted (1 : 50) in fresh LB medium, containing 100 *μ*g/mL ampicillin and 0.04 *μ*M ZnSO_4_ and grown to the optical density (OD) at 600 nm OD_600_ *≈*0.8. The expression of GST-fused CA XII segments was induced with 1 mM IPTG and in the presence of 0.4 mM ZnSO_4_. The culture was grown for 4 h at 30°C and 220 rpm. The cells were harvested, mixed with lysis buffer (20 mM Hepes, 0.1% Triton X-100, 0.15 M NaCl, (pH 8.5), and 1 mM PMSF), and disrupted by sonication. The expression levels of GST-fusion proteins (GST-segment number 1, 37.44 kDa; GST-segment number 2, 37.42 kDa; GST-segment number 3, 38.92 kDa) in the respective lysates of transformed* E. coli* cells were evaluated by sodium dodecyl sulfate-polyacrylamide gel electrophoresis (SDS-PAGE).

### 2.3. Generation of Monoclonal Antibodies

Three 6–8-week-old female BALB/c mice (obtained from a breeding colony at the Department of Immunology of the Center for Innovative Medicine, Vilnius, Lithuania) were immunized by a subcutaneous injection of 50 *μ*g of recombinant CA XII. For an initial immunization, the antigen was emulsified in complete Freund adjuvant (Sigma). Subsequent immunizations at days 28 and 56 were performed without an adjuvant, with the antigen dissolved in PBS, respectively. Antisera were collected two weeks after each injection and tested for the presence of CA XII-specific antibodies by an indirect ELISA. The mouse with the highest antibody titer was boosted subcutaneously with 50 *μ*g of CA XII dissolved in PBS 3 days before the cell fusion. Hybridomas were generated as described by Kohler and Milstein [17]. Mouse splenocytes were fused with Sp2/0-Ag 14 mouse myeloma cells using polyethylene glycol 4000 (PEG, Roth). Hybrid cells were selected in growth medium supplemented with HAT (hypoxanthine, aminopterin, and thymidine) (50x HAT media supplement, Sigma-Aldrich). Culture supernatants from wells with viable clones were screened by an indirect ELISA using recombinant CA XII protein. Stable hybridoma clones secreting CA XII-specific antibodies were obtained after two cloning cycles by a limiting dilution assay. Hybridoma cells were grown in complete Dulbecco's modified Eagle's medium (DMEM, Biochrom) supplemented with 15% fetal bovine serum (FBS, Biochrom), 2 mM L-glutamine, and 200 *μ*g/mL gentamicin. All procedures involving experimental mice were performed under controlled laboratory conditions in strict accordance with the Lithuanian and European legislation.

### 2.4. Indirect ELISA

The specificities of mouse antisera and hybridoma supernatants were investigated by an indirect ELISA. Recombinant CA XII diluted in coating buffer (0.05 M sodium carbonate salt, pH 9.6) to 5 *μ*g/mL was coated on plates (Nerbe) at 50 *μ*L aliquot per well and incubated at 4°C overnight. The coated wells were blocked with 150 *μ*L of 1% BSA solution in PBS for 1 hour at room temperature (RT). Plates were washed two times with PBS-Tween buffer (0.1% Tween 20 in PBS). Antiserum samples or hybridoma growth medium were diluted in PBS-Tween buffer, added to the wells (50 *μ*L/well), and incubated for 1 hour at RT. The plates were rinsed 5 times with PBS-Tween buffer and then incubated with 50 *μ*L of goat anti-mouse IgG conjugated to horseradish peroxidase (HRP) (Bio-Rad) diluted 1 : 5000 in PBS-Tween buffer for 1 hour at RT. The plates were washed 5 times with PBS-Tween buffer. Peroxidase activity was detected using 50 *μ*L of ready-to-use TMB substrate (Sigma) per well. After 10 min of incubation at RT, the reaction was stopped by adding 25 *μ*L aliquot per well of 10% H_2_SO_4_. The optical density (OD) was measured at 450 nm (reference filter 620 nm) using microplate reader (Tecan, Grödig, Austria).

The isotypes of the MAbs were determined by ELISA using the monoclonal antibody isotyping kit (Thermo Fisher Scientific, Vilnius, Lithuania) according to the manufacturer's protocol.

### 2.5. Determination of the Apparent Dissociation Constant (*K*_*d*_)

The apparent dissociation constants (*K*_*d*_) of the MAbs were determined by an indirect ELISA as described previously [18]. Briefly, the MAbs were prepared in concentrations ranging from 1.9 × 10^−13^ M to 3.3 × 10^−8^ M and incubated in the microtiter plates coated with recombinant CA XII. The plates were then incubated with HRP-labelled anti-mouse IgG (Bio-Rad) and developed with TMB substrate. The apparent *K*_*d*_ was calculated from a titration curve and defined as a molar concentration of the MAbs corresponding to the midpoint between maximum OD_450_ value and the background.

### 2.6. Sodium Dodecyl Sulfate-Polyacrylamide Gel Electrophoresis

Before sodium dodecyl sulfate-polyacrylamide gel electrophoresis (SDS-PAGE), protein samples were added to the Line Marker Reducing sample buffer (Thermo Scientific) and boiled for 5 min. Protein samples (1 *μ*g per lane) were separated by electrophoresis on 12% polyacrylamide gel. The gels were visualized by staining with Coomassie brilliant blue (Sigma).

### 2.7. Immunoblotting

After SDS-PAGE, proteins were transferred to a polyvinylidene difluoride (PVDF) membrane (Roth). The membranes were blocked with 5% milk powder in PBS for 1 h at RT. The membranes were incubated with undiluted hybridoma supernatants for 1 h at RT, followed by incubation with goat anti-mouse IgG conjugated to horseradish peroxidase (HRP) (Bio-Rad) diluted 1 : 4000 in PBS-Tween buffer. The enzymatic reaction was developed using tetramethylbenzidine (TMB) ready-to-use chromogenic substrate (Sigma).

### 2.8. Flow Cytometry Analysis

The reactivities of the MAbs with cellular CA XII were investigated by flow cytometry using 5 human cell lines: A-498 (human kidney carcinoma), U-87 (human primary glioblastoma), A-549 (human lung adenocarcinoma), HeLa (human cervical carcinoma), and CaSki (human cervical carcinoma) (ATCC, Manassas, VA, USA). As a negative control, Chinese hamster ovary (CHO) cells were used. Cells were cultivated in RPMI-1640 growth medium (Biochrom, Berlin, Germany) supplemented with 10% fetal bovine serum (Biochrom), 2 mM L-glutamine, and 200 *μ*g/mL gentamicin in humidified atmosphere at 37°C and 5% CO_2_ to approximately 70% confluence. Harvested cells (10^6^ cells per test) were fixed using a buffer containing paraformaldehyde, washed with BD Perm/Wash buffer (BD Biosciences), and incubated with hybridoma growth medium at 4°C for 30 min. As a negative control, irrelevant MAbs of IgG1 and IgG2a isotypes were used. Binding of the antibodies was determined using FITC-conjugated goat anti-mouse IgG (BD Pharmingen, Franklin Lakes, USA) and measured by standard flow cytometry (FACS) with CyFlowRspace flow cytometer (Partec, Muenster, Germany). Not less than 20000 events per test were evaluated with FloMax 2.7 software.

### 2.9. Immunohistochemistry Analysis

Immunohistochemical staining was performed on formalin-fixed and paraffin-embedded samples of colon adenoma, colon carcinoma, renal carcinoma and normal colon, and kidney tissues at the Institute of Biomedical Technology, University of Tampere (Tampere, Finland). Tissue specimens were collected and tested for CA XII expression as described previously [19]. Tissue sections (approximately 5 *μ*m thick) were stained using an automated Lab Vision Autostainer 480 (LabVision Corporation, Fremont, CA, USA) and Power Vision Poly-HRP Immunohistochemistry kit (ImmunoVision Technologies, Burlingame, CA, USA) according to the manufacturer's protocol. Samples were deparaffinized in xylene and rehydrated in graded alcohols. Tissue sections were incubated in 3% H_2_O_2_ for 5 min and blocked with cow colostrum diluted 1 : 2 in Tris-buffered saline containing 0.05% Tween-20 for 30 min at RT. Slides were incubated with the MAbs for 30 min. After rinsing in wash buffer for 35 min, samples were incubated in poly-HRP-conjugated anti-rabbit/mouse IgG (ImmunoVision Technologies) for 30 min. Slides were visualized using 3,3-o-diaminobenzidine tetrahydrochloride (DAB) ready-to use solution (ImmunoVision Technologies).

## 3. Results 

### 3.1. Generation of Hybridomas Producing MAbs against Recombinant CA XII

In order to generate hybridomas, BALB/c mice were immunized with recombinant CA XII expressed in* E. coli* and purified by affinity chromatography. Recombinant CA XII represents the N-terminal catalytic domain (aa 30–291) of CA XII and lacks the signal, transmembrane, and cytoplasmic domains. To evaluate the immunogenicity of the recombinant CA XII, antiserum specimens were collected after each injection and tested for the presence of CA XII-specific antibodies by an indirect ELISA. After 3 immunizations, the titers of CA XII-specific IgG antibodies in the sera of immunized mice ranged from 1 : 9000 to 1 : 21000 (data not shown). Thus, recombinant CA XII was immunogenic in mice. Spleen cells of the mouse with the highest antibody titer were fused with mouse myeloma cells following standard procedures. At the 12th day after cell fusion, hybrid clones secreting CA XII-specific antibodies were screened by an indirect ELISA on plates coated with the recombinant CA XII. Seven stable hybridoma cell lines producing CA XII-specific MAbs of IgG isotype were generated (Table 2). Six out of 7 MAbs were of IgG1 subtype; one MAb (clone 15A4) was of IgG2a subtype. The hybridomas were cultivated in culture and the supernatants were collected for further characterization of the MAbs.

### 3.2. Specificity and Affinity of the MAbs

**Figure 1:**
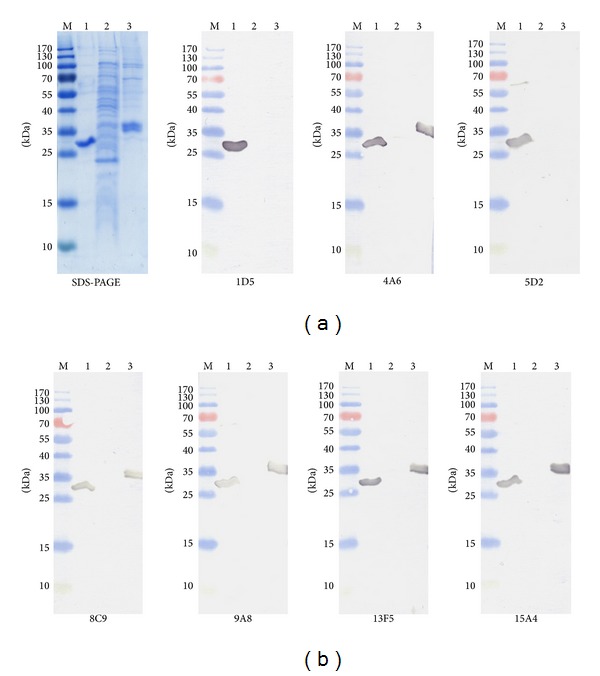
Reactivity of the MAbs with denatured recombinant CA XII expressed in* E. coli* and mammalian cells. Upper-leftmost panel: SDS-PAGE; remaining panels: immunoblot with MAbs 1D5, 4A6, 5D2, 8C9, 9A8, 13F5, and 15A4. Line M: prestained MW markers (Thermo Fisher Scientific, Vilnius); line 1: purified recombinant CA XII proteinexpressed in* E. coli*; line 2: lysate of* E. coli* Rosetta (DE3) strain cells; line 3: purified recombinant CA XII protein expressed in mammalian cells.

All MAbs were reactive in ELISA with the recombinant* E. coli*-expressed CA XII (data not shown). To test the reactivity of the MAbs with SDS-denatured antigen, the purified CA XII protein and crude lysate of* E. coli* transformed with pET21a-CA XII vector were denatured by boiling in sample buffer (Thermo Fisher Scientific) containing SDS and 2-mercaptoethanol and subjected to protein electrophoresis and immunoblotting. All MAbs specifically recognized protein band of 31 kDa, which corresponded to recombinant CA XII (Figure 1, lane 1) and did not cross-react with other proteins of* E. coli* lysates (Figure 1, lane 2). Thus, all MAbs were reactive with SDS-denatured recombinant CA XII (Table 2).

As the MAbs were raised against* E. coli*-expressed recombinant CA XII that did not undergo posttranslational modifications, the ability of the MAbs to recognize glycosylated form of CA XII was evaluated. For this purpose, the reactivities of the MAbs with recombinant CA XII expressed in human HEK 293 cells were tested both by ELISA and Western blot. The MAbs differed in their capability to recognize glycosylated CA XII expressed in mammalian cells. MAbs 4A6, 9A8, and 15A4 showed a strong reactivity with the recombinant glycosylated CA XII both by ELISA and Western blot (Figure 1, lane 3, Table 2). In contrast, MAbs 1D5 and 5D2 were nonreactive with CA XII expressed in mammalian cells (Table 2). MAbs 8C9 and 13F5 showed a moderate reactivity with CA XII expressed in mammalian cells (Figure 1, lane 3, Table 2).

To investigate the cross-reactivities of the MAbs with CA isoforms other than XII, the previously purified CAs were used [15]. The analysis of MAb reactivities both by ELISA and Western blot revealed that 6 MAbs reacted exclusively with the recombinant CA XII and did not show any reactivity with recombinants CA I, CA II, CA VII, and CA XIII (Table 2). Only the MAb 13F5 showed a weak cross-reactivity with CA II and CA VII in ELISA but not in Western blot (Table 2).

To determine the affinity of the MAbs, their apparent *K*_*d*_ values were measured by an indirect ELISA. The *K*_*d*_ values of the MAbs calculated from three experiments ranged from 1.07 × 10^−9^ M to 6.17 × 10^−10^ M, indicating high-affinity binding (Table 2).

### 3.3. Localization of MAb Epitopes

**Figure 2:**
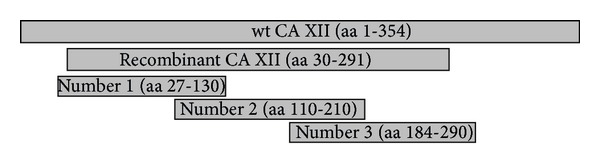
Schematic representation of GST-fused CA XII fragments used for epitope mapping. Wt CA XII: full length CA XII. Recombinant CA XII-*E. coli*-expressed extracellular catalytic domain of CA XII used for the generation and characterization of MAbs.

To identify the epitopes of recombinant CA XII recognized by the MAbs, three partially overlapping GST-fused fragments of CA XII were constructed: fragment number 1 (aa 27–130), fragment number 2 (aa 110–210), and fragment number 3 (aa 184–290). Schematic representation of GST-fused CA XII fragments is shown in Figure 2.

**Figure 3:**
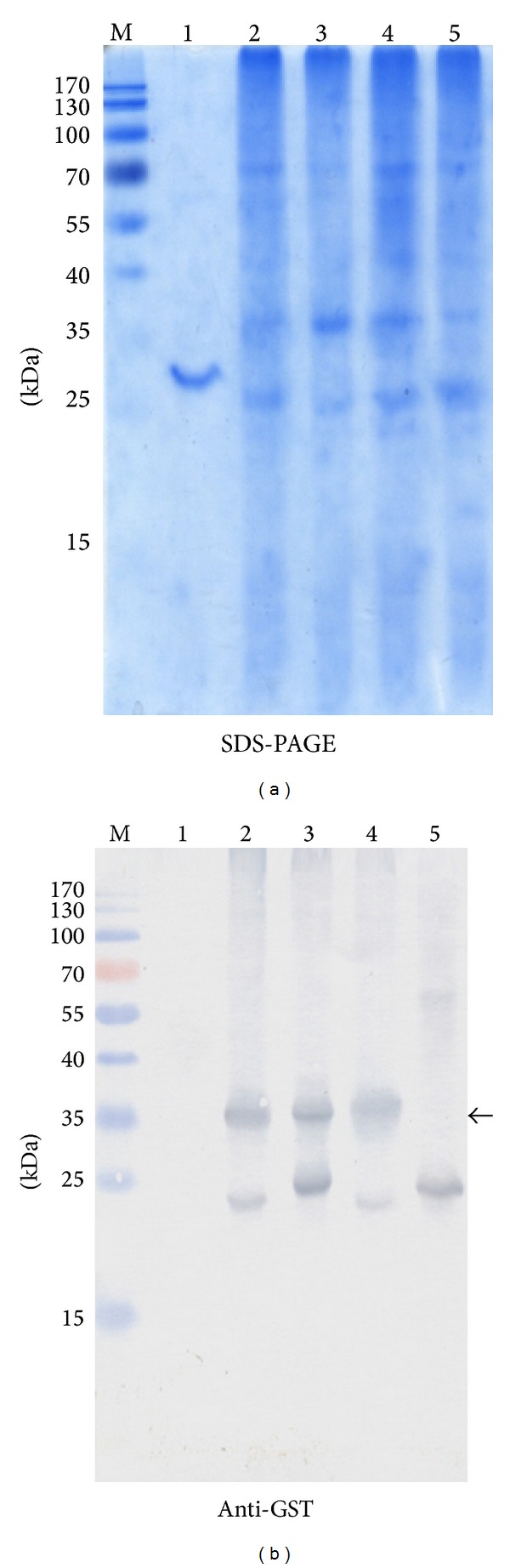
Analysis of the expression of GST-fused CA XII fragments in* E. coli* lysates. (a) SDS-PAGE. (b) Western blot with anti-GST MAbs (Thermo Scientific): lane M: prestained MW markers (Thermo Scientific); lane 1: purified recombinant CA XII protein; lanes 2–4: lysates of transformed* E. coli* cells expressing CA XII fragments number 1 (lane 2), number 2 (lane 3), and number 3 (lane 4); line 5: lysate of transformed* E. coli* cells expressing GST. Arrow (←) indicates GST-fused CA XII fragments.

GST-fused fragments of CA XII were expressed in* E. coli* BL21 (DE3) strain. Expression levels of GST-fused CA XII fragments in* E. coli* lysates were analysed by SDS-PAGE and Western blot using commercial antibodies against GST (Figure 3). All fragments were efficiently expressed in transformed* E. coli* cells. The reactivities of the MAbs with GST-fused CA XII fragments were investigated by Western blot using lysates of transformed* E. coli* cells expressing the respective fragments. MAbs 1D5, 4A6, and 5D2 recognized fragment number 1 that represents the N-terminal region of CA XII (aa 27–130). As the MAbs 1D5, 4A6, and 5D2 were reactive with the whole recombinant catalytic domain of CA XII (aa 30–291) and did not recognize fragment number 2 (aa 111–210), the epitopes for these MAbs were located between aa 30 and 110 of CA XII. MAbs 8C9 and 13F5 reacted exclusively with fragment number 3 that represents C-terminal region of CA XII (aa 184–290). As the MAbs 8C9 and 13F5 did not recognize an overlapping fragment number 2 (aa 111–210), the epitopes for these MAbs were located between aa 210 and 290 of CA XII. MAbs 9A8 and 15A4 recognized both fragment numbers 2 and 3. It was concluded that their epitopes are located between aa 184 and 210, as this is an overlapping sequence of both fragment numbers 2 and 3. Summarized data on MAb reactivities with GST-fused CA XII fragments are presented in Table 3.

### 3.4. The Reactivities of the MAbs with the Cellular CA XII by Flow Cytometry

**Figure 4:**
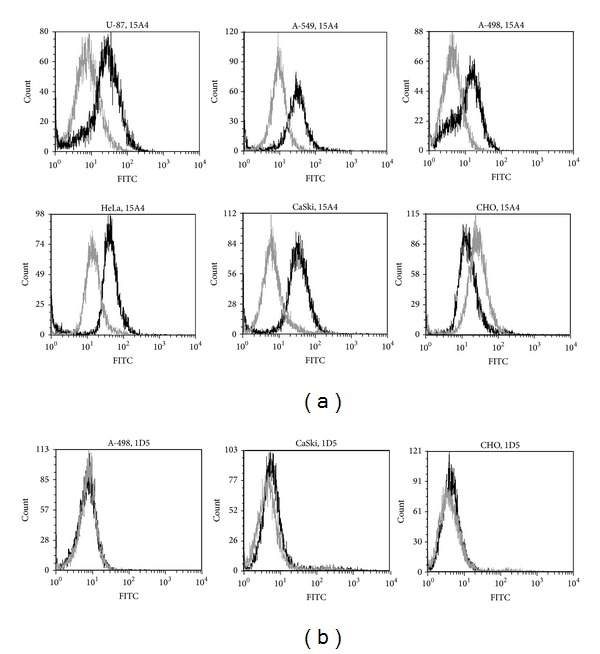
Flow-cytometry analysis of U-87, A-549, A-498, HeLa, and CaSki cell lines immunostained with the MAbs: (a) 15A4 (IgG2a subtype) (black line); (b) 1D5 (IgG2a subtype) (black line); irrelevant MAbs of IgG1 or IgG2a subtypes were used as negative control (gray line).

To investigate the reactivities of the MAbs with the cellular full-length CA XII, we have used human cancer cell lines A-498 (human kidney carcinoma), U-87 (human primary glioblastoma), A-549 (human lung adenocarcinoma), HeLa (human cervical carcinoma), and CaSki (human cervical carcinoma) with previously reported different expression levels of CA XII [13, 20]. As a negative control, Chinese hamster ovary (CHO) cells were used. The cells were cultivated under normoxic conditions, then treated with the MAbs and analysed by flow cytometry. The MAbs differed in their capacity to recognize the cellular CA XII in human cancer cell lines. The MAb 15A4 showed a strong specific immunostaining of A-498, U-87, A-549, CaSki, and HeLa cells and had no reaction with CHO cells used a as a negative control (Figure 4(a)). In contrast, no reactivity of the MAbs 1D5, 4A6, 5D2, and 9A8 with these cell lines was observed (Figure 4(b)).The MAbs 8C9 and 13F5, however, were reactive both with human cell lines and CHO cells (data not shown), indicating nonspecific staining.

### 3.5. The Reactivities of the MAbs with the Cellular CA XII by Immunohistochemistry

**Figure 5:**
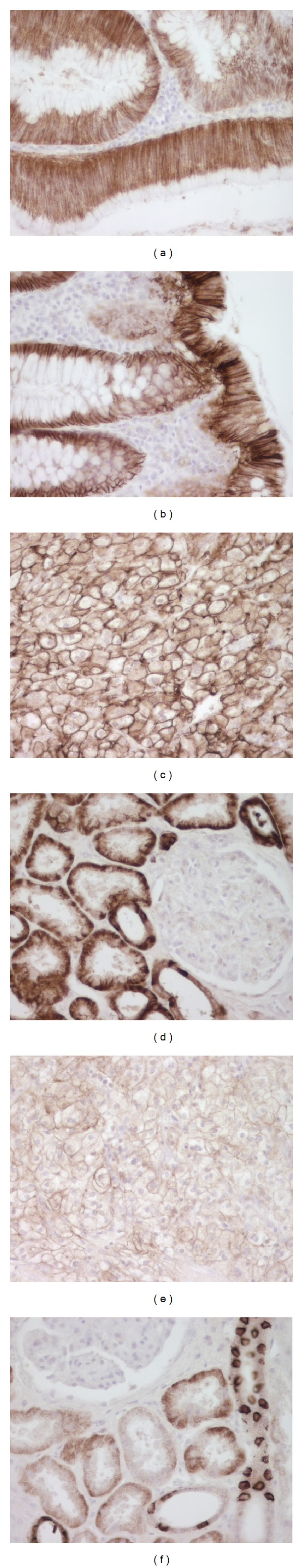
Immunostaining of colon adenoma (a), normal colon (b), normal kidney ((d), (f)), and renal carcinoma ((c), (e)) specimens for CA XII expression using newly produced mAbs 15A4 ((a)–(d)) and 4A6 ((e), (f)). MAb clone 15A4 hybridoma supernatant was diluted 1 : 100; 4A6-1 : 10. Original magnifications ×400.

Immunohistochemistry analysis (IHC) was used to investigate the reactivities of the MAbs with the cellular full-length CA XII protein on formalin-fixed and paraffin-embedded samples of colon adenoma, colon carcinoma, renal carcinoma, and normal colon and kidney tissues. Two MAbs, clones 4A6 and 15A4, showed specific immunostaining of renal carcinoma, colon adenoma, and colon carcinoma specimens and did not show any unspecific background staining of the respective normal tissues (Figure 5). The MAb 4A6 was reactive in IHC at higher concentrations (dilution of hybridoma supernatant 1 : 10, Figures 5(e) and 5(f)) as compared to the MAb 15A4 (dilution of hybridoma supernatant 1 : 100, Figures 5(a)–5(d)), which is explained by different affinities of the MAbs: *K*_*d*_ were 1.07 × 10^−9^ and 2.02 × 10^−10^, respectively. Other MAbs did not show any specific immunostaining of tissue specimens (data not shown). Thus, the IHC results demonstrate the potential of the MAbs 4A6 and 15A4 as specific reagents for the immunodetection of CA XII in formalin-fixed and paraffin-embedded tissue specimens.

## 4. Discussion

CA XII is a single-pass transmembrane protein with an extracellular catalytic domain [1]. Based on its role in tumor progression, this enzyme is considered to be a useful diagnostic and prognostic biomarker for different tumors [6]. However, the data on CA XII distribution in normal and tumor tissues are still incomplete. Investigation of CA XII expression in different cell types might be promoted by the availability of highly specific and well-characterized MAbs. To generate MAbs against membrane-bound proteins, such as carbonic anhydrases, immunizations with intact cells, or cell lysates containing target protein, has been proven useful. The first MAb against CA IX was generated after immunization of mice with human renal cell carcinoma cells [21]. The MAbs raised against cellular CA IX have been employed for the identification of CA IX in renal cell carcinoma and other tumor cells, as well as targeting tumor cells by an enhanced internalization of antibody-bound CA IX [9]. Based on these investigations and other studies confirming the importance of CA IX in tumor development, chimeric MAb G250 against CA IX has been generated and evaluated in a clinical trial for tumor-specific therapy [22]. Recently, two research groups generated MAbs against CA XII after immunization either with A549 cell lysate or live cells, respectively [13, 14]. Selection of positive hybridomas after immunization of rats with live A549 lung cancer cells was performed by flow cytometry using intact A549 cells. The MAb 6A10 raised against cellular CA XII was shown to inhibit CA XII enzymatic activity and growth of tumor cells* in vitro* [14].

In the current study, we have applied another approach to generate CA XII-specific MAbs. As an immunogen, we have used the recombinant* E. coli*-expressed catalytic domain of CA XII. Immunization with the recombinant antigen was successful and resulted in a panel of CA XII-specific MAbs that differed in their cross-reactivities with other CA isoforms as well as their ability to recognize cellular CA XII or mammalian cell-derived recombinant CA XII. Six out of 7 newly developed MAbs were reactive exclusively with CA XII and did not recognize other CA isoforms used in this study: CA I, CA II, CA VII, and CA XIII. Five out of 7 MAbs were reactive with recombinant CA XII expressed in HEK 293 cells, which differs from the* E. coli*-expressed protein according to its glycosylation pattern. The reactivity of the MAbs with glycosylated CA XII is in line with epitope mapping data performed within the current study. According to the* UniProt* database, glycosylation sites in CA XII are located at Asn-28, Asn-80, and Asn-162 [23]. The MAbs 1D5 and 5D2, directed against N terminally located epitope (aa 30–110), did not recognize mammalian cell-derived CA XII, while the MAb 15A4 directed against the aa 192–205 sequence of CA XII not containing potential glycosylation sites was strongly reactive with mammalian cell-derived recombinant CA XII both by ELISA and Western blot. The ability of the MAb 15A4 to recognize properly modified cellular CA XII was also confirmed by flow cytometry as this MAb showed a strong specific immunostaining of A-498, U-87, A-549, CaSki, and HeLa cells expressing CA XII. Moreover, the MAb 15A4 recognized cellular CA XII protein by IHC on formalin-fixed and paraffin-embedded specimens of renal carcinoma, colon adenoma, and colon carcinoma as well as normal kidney and colon tissues. These data demonstrate the potential of the MAb 15A4 as a highly specific reagent for studying CA XII expression in tumor and normal tissues.

## 5. Conclusions

Our approach to use the recombinant* E. coli*-expressed catalytic domain of CA XII as an immunogen was successful and resulted in highly specific MAbs reactive with cellular CA XII. The well-characterized MAbs, in particular clone 15A4, represent a promising diagnostic tool for the immunodetection of CA XII in biological specimens.

## Figures and Tables

**Table 1:** PCR primers used to produce three overlapping segments of CA XII.

Segment number	Segment sequence	PCR primer sequences*	Restriction endonuclease
#1	Val 27-Gly130	5′ CCCGGGATCCGTGAACGGTTCCAAG 3′	BamHI
		5′ ACGGCGGCCGCTTAGCCGTGCGGGTCATT 3′	NotI

#2	Ser111-Leu210	5′ GGGCGGATCCTCTCGCTACAGTGCC 3′	BamHI
		5′ GTCCTCTCGAGTTACAGCTCTTCAATGTTG 3′	XhoI

#3	Asp184-Ser290	5′ TCCGGGATCCGACAAGATCTTCAGTC 3′	BamHI
		5′ AGACCTCGAGTTAGGAGAAGGAGGTGTATAC 3′	XhoI

*Restriction endonuclease recognition sites are underlined.

**Table 2:** Characterization of the MAbs raised against recombinant CA XII: summarized data.

							Reactivity with CA XII:					
MAb clone	Cross-reactivity of the MAbs with recombinant CA isoforms				IgG subtype	Apparent *K*_*d*_, *M*	expressed in* E. coli *		expressed in mammalian cells		in cancer cells or tissue	
	CA I	CA II	CA VII	CA XIII			ELISA	WB	ELISA	WB	FACS	IHC
1D5	−	−	−	−	IgG1	2.07 × 10^−10^	+	+	−	−	−	−
4A6	−	−	−	−	IgG1	1.07 × 10^−9^	+	+	+	+	−	−/+
5D2	−	−	−	−	IgG1	5.45 × 10^−10^	+	+	−	−	−	−
8C9	−	−	−	−	IgG1	1.88 × 10^−10^	+	+	−/+	+	−	−
9A8	−	−	−	−	IgG1	6.17 × 10^−10^	+	+	+	+	−	−
13F5	−	−/+	−/+	−	IgG1	1.12 × 10^−10^	+	+	−/+	+	−	−
15A4	−	−	−	−	IgG2a	2.02 × 10^−10^	+	+	+	+	+	+

“+”: strong reaction, “−/+”: weak reaction, “−”: no reaction.

**Table 3:** The reactivity of the MAbs with GST-fused CA XII fragments and predicted localization of MAb epitopes.

MAb clone	CA XII protein fragments			Predicted localization of MAb epitopes
	#1 (aa 27–130)	#2 (aa 111–210)	#3 (aa 184–290)	
1D5	+	−	−	aa 30–110
4A6	+	−	−	aa 30–110
5D2	+	−	−	aa 30–110
8C9	−	−	+	aa 210–290
9A8	−	+	+	aa 184–210
13F5	−	−	+	aa 210–290
15A4	−	+	+	aa 184–210
